# Computed Tomography: Return on Investment and Regional Disparity Factor Analysis

**DOI:** 10.3389/fpubh.2018.00380

**Published:** 2019-01-10

**Authors:** Shinya Imai, Manabu Akahane, Tomoaki Imamura

**Affiliations:** ^1^Faculty of Health Sciences, Butsuryo College of Osaka, Osaka, Japan; ^2^Department of Public Health, Health Management and Policy, y, Nara, Japan

**Keywords:** computed tomography, multi-slice CT, single-slice CT, net profits, SSCT in Japan, MSCT in Japan

## Abstract

The number of computed tomography (CT) systems in operation in Japan is approximately 4.3 times higher than that of the OECD average. However, CT systems are expensive, and thus, a heavy financial burden for hospital management. We calculate the annual net profits from CT introduction in Japan for single-slice CT (SSCT), multi-slice CT (MSCT), number of hospital beds, and prefecture. We also analyze the factors that affect CT profitability. First, the annual income per CT in operation is estimated for 2011. Second, the annual costs per CT are calculated as the sum of depreciation, maintenance, and labor costs. Finally, the annual net profits per CT are estimated for SSCT and MSCT, the number of hospital beds, and prefecture. A correlation analysis between the annual net profits, population, and number of physicians per CT equipment is used to determine the determinants of the net CT profits by prefecture. Our results show that, for hospitals with fewer than 100 beds, the annual net CT profits are higher for SSCT than MSCT, and vice versa for hospitals with at least 100 beds. Both SSCT and MSCT increased profits as the number of hospital beds increased. The annual net CT profits per prefecture are USD −12,105 for SSCT and USD 87,233 for MSCT, on average. The annual net profits per prefecture and population per CT show positive correlations with both SSCT and MSCT, as do the annual net profits per prefecture and number of physicians per CT. Thus, choosing high-performance MSCT is advantageous in terms of profitability in facilities with at least 100 beds. Additionally, CT profitability presumably affects the balance between the number of introduced CTs, population per CT, and number of physicians per CT.

## Introduction

Computed tomography (CT) was invented in 1968 by Godfrey Hounsfield at EMI Corporation, United Kingdom. It now provides immeasurable benefits in the field of medical care. As CT has superior spatial resolution than other examinations, it is excellent for stroke, acute abdominal disorder, and cancer screening, among other disorders (1–4). Additionally, with the advent of multi-slice CT (MSCT) that uses multi-row detectors, it has become possible to photograph thin slices of tissue during a short time period, making, for example, coronary arteries and the colon subject to CT examinations (5–8). Moreover, applying iterative reconstruction has made it possible to obtain high quality images at low doses, that is, screening for lung cancer at a low dose is also possible (4, 9, 10). Consequently, CT has rapidly spread worldwide because of its rapid progress and high diagnostic capability.

Specifically, the spread of CT in Japan has been exceptional. According to 2013 data from the Organization for Economic Co-operation and Development (OECD), the number of CTs per million inhabitants in Japan is 101.3, ~4.3 times the OECD average of 23.6 units (11). As such, it is necessary to assess the effective use and placement of CTs in Japan. Additionally, as a CT system is an expensive medical device, it may be a heavy burden for hospital management (12), especially because, in Japan, there are cases where CT has also been introduced in small hospitals and medical offices. However, to the best of our knowledge, the profitability of CT introduction has not been sufficiently studied to date.

Consequently, we calculate the annual net profits of the CTs in Japan based on performance, number of hospital beds, and prefecture in order to analyze the determinants of CT profitability. The purpose of this study is to clarify the influence of CT on hospital management from the viewpoint of income and costs. CT income and cost data are taken from the Ministry of Health, Labor, and Welfare. Depending on the number of hospital beds, these data show the performance level of CT where the income and cost balance is excessive. Our results provide useful information for hospital administrators when updating CT infrastructure and for hospital management generally.

## Materials and Methods

### CT Profitability by the Number of Hospital Beds

#### Estimation of the Annual CT Income by the Number of Hospital Beds

We calculate annual CT income for the number of beds in each facility by multiplying the annual CT examination numbers by the examination fee of each CT system. The CT performance is divided into single-slice CT (SSCT), with one X-ray detector, and multiple-slice CT (MSCT) with multiple X-ray detectors. Facilities are categorized by the number of hospital beds as follows: 0–19, 20–49, 50–99, 100–199, 200–299, 300–499, and 500 beds and above. The number of annual CT examinations is estimated by the performance and number of hospital beds, using the total number of CT systems implemented and total number listed in the Survey of Medical Institutions of 2011 (13). The income per CT examination is estimated using the medical treatment fees in Japan in 2011. From these medical treatment fees, we understand the remuneration that medical institutions and pharmacies receive from insurers as compensation for insured medical services. The fees corresponding to each item are added for each medical procedure carried out, after which, the total fees are calculated.

Based on these figures, the annual income per CT scanner is calculated for each procedure and prefecture by multiplying the number of examinations per CT scanner by the income per CT examination. The income per CT examination is calculated for each procedure according to the imaging, contrast enhancement, diagnosis, electronic image management, and radiology diagnosis fees 1 or 2 (see Table 1). As of April 2, 2018, 1 dollar was equal to 105.89 yen.

**Table 1:** Medical fees.

**Medical fees**		**(USD)**
Imaging fee	SSCT	56.7
	MSCT (Fewer than 16 detector rows)	77.4
	MSCT (More than 16 detector rows)	85.0
Contrast-enhanced fee		47.2
Diagnostic fee		42.5
Electronic imaging management		11.3
Radiological diagnosis fee I		6.6
Radiological diagnosis fee II		17.0

*SSCT, single-slice computed tomography; MSCT, multi-slice computed tomography*.

#### Estimation of the Annual CT Costs by the Number of Hospital Beds

Annual CT costs are calculated as the sum of depreciation expenses for the main unit, maintenance costs, and labor costs. Personnel expenses are calculated using the Osaka Prefectural Public Hospital Questionnaire and 2011 Basic Survey on Wage Structure (14). These are estimated for each procedure and prefecture using a CT cost model (Table 2) and the number of CT scanners. The depreciation expenses for the main unit are calculated using a linear method with the main unit price and assuming an amortization period of 6 years. The maintenance fee is set as the total annual maintenance fee, including periodic inspections and repair costs. The number of CTs is calculated using the number of CTs for each hospital bed in Tokyo, Gunma, Nara, Kochi, and Tottori (Table 3). The large dataset of CTs makes it difficult to investigate the number of CTs for large (e.g., Tokyo), medium (e.g., Gunma, Nara), and small cities (e.g., Kochi, Tottori). Labor costs are estimated using the average number of doctors, medical radiology technicians, and nurses necessary for CT examinations in Osaka prefectural public hospitals, as well as the average numbers of examinations and CT systems by prefecture.

**Table 2:** CT cost model.

**Performance**		**Unit price (USD)**	**Depreciation (USD)**	**Maintenance cost (USD)**	**Total cost (USD)**
SSCT		188,875	31,479	28,331	59,810
MSCT	Fewer than four detector rows	283,313	47,219	66,106	113,325
	Four to Fewer than 16 detector rows	377,750	62,958	75,550	138,509
	16 to fewer than 64 detector rows	661,063	110,177	141,656	251,834
	More than 64 detector rows	1,416,564	236,094	188,875	424,969

*SSCT, single-slice computed tomography; MSCT, multi-slice computed tomography*.

**Table 3:** Annual CT income and costs by the number of hospital beds.

**Performance**	**Number of hospital beds**	**Examination number per CT**	**Fee per CT examination (USD)**	**Annual income (USD)**	**Depreciation Maintenance costs (USD)**	**Labor costs (USD)**	**Annual costs (USD)**
SSCT	0–19	381	115	43,583	59,810	8,815	68,626
	20–49	587	118	69,358	59,810	13,595	73,406
	50–99	672	118	79,566	59,810	15,562	75,372
	100–199	688	123	84,969	59,810	15,938	75,748
	200–299	536	132	70,478	59,810	12,405	72,215
	300–499	736	138	101,394	59,810	17,053	76,863
	more than 500	1,008	144	145,185	59,810	23,350	83,161
MSCT	0–19	850	139	117,818	172,414	19,683	192,097
	20–49	1,165	142	165,647	178,251	26,987	205,238
	50–99	1,620	143	232,335	201,340	37,521	238,861
	100–199	2,544	149	380,013	215,680	58,921	274,601
	200–299	4,055	159	643,489	240,061	93,914	333,976
	300–499	6,058	165	999,729	262,935	140,299	403,234
	more than 500	7,818	171	1,340,313	281,102	181,056	462,159

*SSCT, single-slice computed tomography; MSCT, multi-slice computed tomography*.

#### Estimation of the Annual Net CT Profits by the Number of Hospital Beds

Annual net profits per CT scanner are calculated for each procedure and number of hospital beds using the annual income and costs per CT scanner.

### CT Profitability by Prefecture

#### Estimation of the Annual CT Income by Prefecture

Annual CT income is calculated for each prefecture and CT by multiplying the number of annual examinations by the income from each CT examination. The number of examinations per CT per prefecture is estimated using the total number of CT implementations and total numbers listed in the Survey of Medical Institutions of 2011. The income per CT examination is calculated for each procedure according to the imaging, contrast enhancement, diagnosis, electronic image management, and radiologic diagnosis fees 1 or 2 (see Table 1).

#### Estimation of the Annual CT Costs by Prefecture

Annual CT costs are calculated for each CT and prefecture using a CT cost model similar to the estimation method by the number of hospital beds. The CT numbers by prefecture incorporate the MSCTs listed in the Data Book of Medical Devices & Systems 2013 (15).

#### Estimation of the Annual Net CT Profits by Prefecture

Annual net profits per CT scanner are calculated for each procedure and prefecture using the annual income and costs per CT scanner.

### Factor Analysis on the Annual Net CT Profits by Prefecture

A correlation analysis is conducted to investigate the relationship between the annual net CT profits by prefecture, population per CT, and number of doctors per CT. The population per CT is calculated by prefecture using the population listed in the Population Estimates of 2011 (16), while the number of physicians per CT is calculated using the number of physicians listed in the Survey of Physicians, Dentists and Pharmacists of 2012 (17).

## Results

### CT Profitability by the Number of Hospital Beds

#### Estimation of the Annual CT Income by the Number of Hospital Beds

The fees per CT examination by the number of hospital beds show an increasing trend for both SSCT and MSCT as the number of hospital beds increases. Although the number of examinations per CT scanner increases as the number of hospital beds increases, the tendency is particularly strong for MSCT. The annual revenue per CT is USD 43,583–145,185 for SSCT and USD 117,818–1,340,313 for MSCT. The annual income for MSCT is higher for all numbers of hospital beds, and both SSCT and MSCT show an increasing trend as the number of hospital beds increases (Table 3).

#### Estimation of the Annual CT Costs by the Number of Hospital Beds

The total CT depreciation and maintenance expenses by the number of hospital beds are USD 59,810 for SSCT and USD 172,414–281,102 for MSCT. Personnel expenses are USD 8,815–23,350 for SSCT and USD 19,683–181,056 for MSCT. Both SSCT and MSCT increase with the number of hospital beds, but the tendency is stronger for MSCT. The annual cost per CT is estimated at USD 68,626–83,161 for SSCT and USD 192,097–462,159 for MSCT. The annual costs for MSCT are higher for all numbers of hospital beds, and both SSCT and MSCT show an increasing tendency as the number of hospital beds increases (Table 3).

#### Estimation of the Annual Net CT Profits by the Number of Hospital Beds

**Figure 1:**
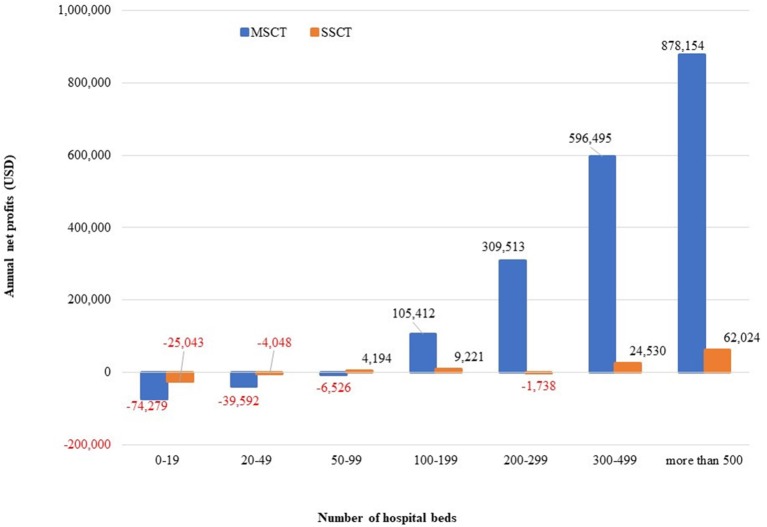
Annual net CT profits by the number of hospital beds.

The annual net profits per CT by the number of hospital beds are USD −25,043 to 62,024 for SSCT and USD −74,279 to 878,154 for MSCT. SSCT shows a higher profit for hospitals below 100 beds, while MSCT shows a higher tendency for hospitals with at least 100 beds. Nevertheless, both SSCT and MSCT show an increasing tendency as the number of hospital beds increases (Figure 1).

### CT Profitability by Prefecture

#### Estimation of the Annual CT Income by Prefecture

The annual income per SSCT by prefecture is estimated to range from approximately USD 38,360 to 98,886, while the annual income per MSCT by prefecture is estimated to range from USD 277,958 to 600,490 per prefecture (Table 4).

**Table 4:** Annual net CT profits by prefecture.

**Prefectures**	**Number of CT (unit)**		**Annual income (USD)**		**Annual cost (USD)**		**Annual net profits (USD)**	
	**SSCT**	**MSCT**	**SSCT**	**MSCT**	**SSCT**	**MSCT**	**SSCT**	**MSCT**
Hokkaido	299	514	81,640	401,362	78,740	341,262	2,900	60,100
Aomori	98	105	52,472	332,376	71,991	317,480	−19,519	14,896
Iwate	73	99	61,072	325,719	73,981	302,462	−12,909	23,257
Miyagi	51	107	62,830	484,085	74,388	369,533	−11,558	114,552
Akita	39	58	84,725	477,326	79,454	351,645	5,271	125,681
Yamagata	40	71	98,886	504,055	82,731	362,363	16,155	141,692
Ibaraki	97	206	50,002	405,071	71,419	339,596	−21,417	65,475
Tochigi	82	134	48,789	381,344	71,139	330,828	−22,350	50,516
Gumma	68	149	49,396	363,517	71,279	333,843	−21,883	29,673
Saitama	168	375	82,847	439,904	79,019	341,046	3,827	98,858
Chiba	111	301	66,655	546,237	75,273	380,500	−8,618	165,737
Tokyo	273	771	75,384	513,917	77,293	371,510	−1,908	142,407
Kanagawa	186	379	85,273	573,968	79,581	382,055	5,692	191,914
Niigata	106	133	62,625	491,118	74,340	353,869	−11,716	137,249
Toyama	72	75	41,642	500,449	69,485	381,549	−27,843	118,900
Ishikawa	51	88	60,901	484,272	73,941	357,270	−13,040	127,002
Fukui	38	71	55,641	384,049	72,724	315,019	−17,083	69,029
Yamanashi	29	52	93,532	399,833	81,492	337,143	12,040	62,690
Nagano	67	156	81,807	447,185	78,779	341,346	3,029	105,839
Gifu	116	127	50,535	533,854	71,543	365,959	−21,007	167,894
Shizuoka	143	214	54,633	483,327	72,491	348,794	−17,858	134,534
Aichi	236	371	64,328	600,490	74,734	391,935	−10,406	208,556
Mie	100	93	52,344	563,042	71,961	394,928	−19,618	168,114
Shiga	27	70	68,535	577,821	75,708	398,084	−7,173	179,737
Kyoto	53	156	74,071	560,469	76,989	391,981	−2,918	168,488
Osaka	254	550	75,957	522,819	77,425	361,193	−1,468	161,626
Hyogo	188	370	68,975	462,447	75,809	357,625	−6,835	104,822
Nara	31	87	55,662	527,058	72,729	382,284	−17,067	144,773
Wakayama	67	99	50,343	398,680	71,498	330,218	−21,155	68,462
Tottori	25	50	56,654	469,409	72,959	349,799	−16,305	119,609
Shimane	23	58	69,320	429,558	75,889	347,199	−6,569	82,359
Okayama	84	187	56,198	380,120	72,853	326,420	−16,655	53,699
Hiroshima	137	237	59,510	375,147	73,620	322,812	−14,109	52,336
Yamaguchi	99	129	54,671	375,691	72,500	321,414	−17,829	54,277
Tokushima	69	98	38,360	277,958	68,726	284,892	−30,366	−6,934
Kagawa	77	93	56,480	382,089	72,918	342,736	−16,438	39,353
Ehime	86	129	49,698	422,764	71,349	344,898	−21,651	77,867
Kochi	64	92	41,235	287,891	69,391	305,542	−28,156	−17,651
Fukuoka	239	365	71,394	429,842	76,369	352,738	−4,975	77,104
Saga	40	82	49,970	315,952	71,412	307,777	−21,442	8,174
Nagasaki	81	121	73,779	426,393	76,921	348,677	−3,142	77,716
Kumamoto	106	195	48,526	305,410	71,078	302,632	−22,552	2,779
Oita	65	152	46,174	331,384	70,534	317,059	−24,360	14,326
Miyazaki	74	99	67,801	312,876	75,538	321,076	−7,737	−8,199
Kagoshima	131	192	65,979	285,258	75,116	312,057	−9,137	−26,799
Okinawa	34	86	40,187	426,489	69,148	366,257	−28,961	60,232
Mean	100	181	62,118	433,044	74,223	345,811	−12,105	87,233

*SSCT, single-slice computed tomography; MSCT, multi-slice computed tomography*.

#### Estimation of the Annual CT Costs by Prefecture

The annual costs per SSCT by prefecture are estimated to range from USD 68,726 to 82,731, while the annual costs per MSCT by prefecture range from USD 284,892 to 398,084 (Table 4).

#### Estimation of the Annual Net CT Profits by Prefecture

The annual net profits per SSCT by prefecture range from USD −30,366 to 16,155, with the average deficit being approximately USD −12,105. The annual net profits per MSCT by prefecture range from USD −26,799 to 208,556, with an average surplus of USD 87,233. As shown in Table 4, the annual net profits of SSCT are in deficit in most prefectures, while those of MSCT are in surplus in most prefectures.

### Factor Analysis of the Annual Net CT Profits by Prefecture

We find a positive correlation between the annual net profits per SSCT by prefecture and population per CT (*r* = 0.481, *P* < 0.01), and also between the annual net profits per MSCT by prefecture and population per CT (*r* = 0.764, *P* < 0.01). Similarly, we find a positive correlation between the annual net profits per SSCT by prefecture and number of physicians per CT (*r* = 0.438, *P* < 0.01), and also between the annual net profits per SSCT by prefecture and number of physicians per CT (*r* = 0.683, *P* < 0.01) (Table 5). We observe that profits tend to increase as the population per CT increases.

**Table 5:** Factor analysis of the balance of annual net profits.

**Prefectures**	**Annual net profits (USD)**		**Population per CT (thousand people/unit)**	**Physician per CT (people/unit)**
	**SSCT**	**MSCT**	
Hokkaido	2,900	60,100	6.7	15.8
Aomori	−19,519	14,896	6.7	13.0
Iwate	−12,909	23,257	7.6	15.1
Miyagi	−11,558	114,552	14.7	33.9
Akita	5,271	125,681	11.1	23.8
Yamagata	16,155	141,692	10.5	23.4
Ibaraki	−21,417	65,475	9.8	17.1
Tochigi	−22,350	50,516	9.3	19.9
Gumma	−21,883	29,673	9.2	20.5
Saitama	3,827	98,858	13.3	20.5
Chiba	−8,618	165,737	15.1	26.9
Tokyo	−1,908	142,407	12.6	39.7
Kanagawa	5,692	191,914	16.0	32.4
Niigata	−11,716	137,249	9.9	19.2
Toyama	−27,843	118,900	7.4	18.3
Ishikawa	−13,040	127,002	8.4	23.3
Fukui	−17,083	69,029	7.4	18.1
Yamanashi	12,040	62,690	10.6	23.6
Nagano	3,029	105,839	9.6	21.2
Gifu	−21,007	167,894	8.5	17.1
Shizuoka	−17,858	134,534	10.5	20.3
Aichi	−10,406	208,556	12.2	25.6
Mie	−19,618	168,114	9.6	19.6
Shiga	−7,173	179,737	14.6	31.4
Kyoto	−2,918	168,488	12.6	39.2
Osaka	−1,468	161,626	11.0	29.7
Hyogo	−6,835	104,822	10.0	23.7
Nara	−17,067	144,773	11.8	26.5
Wakayama	−21,155	68,462	6.0	16.7
Tottori	−16,305	119,609	7.8	23.3
Shimane	−6,569	82,359	8.8	24.0
Okayama	−16,655	53,699	7.2	20.7
Hiroshima	−14,109	52,336	7.6	19.5
Yamaguchi	−17,829	54,277	6.3	16.1
Tokushima	−30,366	−6,934	4.7	14.6
Kagawa	−16,438	39,353	5.8	15.9
Ehime	−21,651	77,867	6.6	16.7
Kochi	−28,156	−17,651	4.9	14.3
Fukuoka	−4,975	77,104	8.4	25.1
Saga	−21,442	8,174	6.9	18.1
Nagasaki	−3,142	77,716	7.0	20.1
Kumamoto	−22,552	2,779	6.0	16.7
Oita	−24,360	14,326	5.5	14.6
Miyazaki	−7,737	−8,199	6.5	15.7
Kagoshima	−9,137	−26,799	5.3	13.1
Okinawa	−28,961	60,232	11.7	28.3
Correlation coefficients		SSCT	0.481(*p* < 0.01)	0.438(*p* < 0.01)
		MSCT	0.764(*p* < 0.01)	0.683(*p* < 0.01)

*SSCT, single-slice computed tomography; MSCT, multi-slice computed tomography*.

## Discussion

This study suggests that CT performance and number of hospital beds influence annual net CT profits. The annual net profits per CT show an increase for SSCT and MSCT as the number of hospital beds increases, especially for MSCT. Additionally, annual net profits tend to be high for SSCT, which is less expensive for hospitals below 100 beds, but they are higher for MSCT for hospitals with at least 100 beds. This is because the number of examinations per CT varies greatly depending on the performance and number of hospital beds. As MSCT has a larger number of examinations per CT by number of hospital beds than SSCT, and increases more rapidly with rising bed numbers, the MSCTs introduced into facilities with higher bed numbers have higher annual net profits. Moreover, the income per CT examination varies with the CT performance and number of hospital beds because the diagnostic fee is USD 56.7 for SSCT, USD 77.4 for below 16 detector rows for MSCT, and USD 87.8 for 16 detector rows or more for MSCT. However, MSCT has more than three times the annual depreciation and maintenance costs of SSCT. In facilities with fewer than 100 beds, the annual net profits may be lower for SSCT because the necessary number of examinations cannot be ensured. MSCT has higher diagnostic ability than SSCT, and therefore, we suggest that it is better to introduce MSCT for 100+ bed hospitals (18).

Recently, compelling evidence was reported that a substantial fraction of the ≈80 million annual CT exams in the United States of America are performed without sound medical justification. This quantitative evidence is derived from comparing actual CT use patterns with expected CT utilization provided appropriate clinical decision guidelines are followed (19). Recent studies suggest that if appropriate clinical criteria are followed, 20–40% of CT scans could be avoided (20–22). Similar to Japan, CT use has been increasing in other Asian countries too (23, 24). Hu et al. (24) reported that CT utilization rates increased significantly between 2009 and 2013 in emergency departments. They speculated that CT scans may be used for rapid screening to facilitate patients' disposition rather than to confirm diagnosis, given the stress of emergency department crowding and potential lawsuits. They also suggested further investigation to determine whether increasing CT utilization was efficient and cost-effective. Our study clearly shows that selecting high-performance MSCTs for 100+ bed facilities is advantageous in terms of profitability and hospital management.

The usage of CT scans is high in Japan compared to other countries due to the high number of CTs per million inhabitants of Japan compared to the OECD average (11). Therefore, it is important to determine the effective use and placement of CTs in Japan and to confirm the profitability of CT introduction. In this study, we showed that initial costs to purchase CT equipment and maintenance and running costs are fairly high, especially for MSCT, occasionally placing a heavy financial burden on the hospital management. Therefore, there is a possibility that doctors may recommend CT scans to recover initial and maintenance costs over the short term. However, CTs scan can provide an accurate diagnosis for various diseases, as our study clearly demonstrates that CT utilization rates relate to a reduction in mortality from accidents such as falling, drowning, and asphyxia; this indicates that screening patients with CT in the emergency room, especially with MSCT, has clinical advantages such as reduced mortality (25). Taken together with the results of our previous research, this study finds that CT equipment (especially MSCT) has beneficial effects for both emergency medicine and hospital management.

SSCT has a significant drawback in that only one slice of tissue can be acquired per rotation. Thus, scanning an anatomical range of, for example, 30 cm in length using a 10 mm collimation requires 30 tube rotations. Therefore, when examining a patient, he or she must hold his or her breath for a long time. However, these problems have been solved by introducing MSCT. As the MSCT scanner is equipped with multiple rows of detectors (e.g., 16–256 rows), the examination speed is faster. In addition, it can set the slice width to be thinner than that in SSCT, and thus, it shows good results with superior diagnostic evaluation (26). In addition to the medical benefits to patients, for hospitals with 100 beds or more, the balance of income and costs is likely to yield a surplus, thus generating increased profits. Therefore, it may be possible for hospitals with SSCT to consider updating to MSCT. However, in hospitals with fewer than 100 beds, this move could spell a high possibility of the additional expenses becoming a burden. Thus, hospital management should take account of these considerations.

According to the correlation analysis between the annual net profits per CT by prefecture, population per CT, and number of physicians per CT, we find a positive correlation for both SSCT and MSCT. Therefore, if the population per CT is large, the number of patients that use a CT is likely to increase. However, if the number of physicians per CT is large, the number of instructions for CT examination from each doctor is also likely to increase. These determinants of the increase in the annual number of examinations per CT are considered to be reflected in the annual net CT profits.

Nevertheless, our study is also beset by certain limitations. First, only the MSCT depreciation expenses for the main unit, maintenance costs, and personnel expenses are recorded as expenses that influence the net profit, without including other factors affecting the profitability of the hospital as a whole, such as indirect costs. Second, the main unit and maintenance costs for CT are estimates from a simplified model, and may differ from actual costs. In particular, although the price at the time of purchase may vary considerably depending on the medical institution and purchase time, we considered only a uniform estimate. Third, the high usage of CT scans in Japan is driven by hospital profitability. However, as mentioned earlier, our previous study showed that performing accurate diagnosis using MSCT in emergency medicine could reduce mortality rates in terms of accidental death, indicating its benefits for patients.

In conclusion, by estimating the annual net CT profits of CT, we found that selecting high-performance MSCTs for 100+ bed facilities is advantageous in terms of profitability and hospital management. Additionally, the profitability of CT is affected by the introduction of CT scanners in the area, population, and number of physicians. In this trial calculation, the net profits may turn into deficits depending on the performance of the CTs to be introduced and number of hospital beds. However, decisions on introduction of CT should consider not only profitability, but also the clinical necessity of each medical facility.

## Author Contributions

SI designed the study and wrote the initial draft of the manuscript. MA contributed to the analysis and interpretation of data, and assisted in the preparation of the manuscript. TI contributed to data collection and interpretation, and critically reviewed the manuscript. All authors approved the final version of the manuscript, and agree to be accountable for all aspects of the work in ensuring that questions related to the accuracy or integrity of any part of the work are appropriately investigated and resolved.

### Conflict of Interest Statement

The authors declare that the research was conducted in the absence of any commercial or financial relationships that could be construed as a potential conflict of interest.
